# Surgery in the era of the 'omics revolution

**DOI:** 10.1002/bjs.9722

**Published:** 2015-01-27

**Authors:** A. D. Beggs, M. P. Dilworth

**Affiliations:** ^1^ Translational Surgical Biology LaboratorySchool of Cancer Sciences, University of BirminghamVincent DriveBirmingham B15 2TTUK

## Abstract

**Background:**

Surgery is entering a new phase with the revolution in genomic technology. Cheap, mass access to next‐generation sequencing is now allowing the analysis of entire human genomes at the DNA and RNA level. These data sets are being used increasingly to identify the molecular differences that underlie common surgical diseases, and enable them to be stratified for patient benefit.

**Methods:**

This article reviews the recent developments in the molecular biology of colorectal, oesophagogastric and breast cancer.

**Results:**

The review specifically covers developments in genetic predisposition, next‐generation sequencing studies, biomarkers for stratification, prognosis and treatment, and other 'omics technologies such as metabolomics and proteomics.

**Conclusion:**

There are unique opportunities over the next decade to change the management of surgical disease radically, using these technologies. The directions that this may take are highlighted, including future advances such as the 100 000 Genomes Project.

## Abstract 2

May individualize cancer treatment

## Introduction

The field of molecular biology has undergone rapid advancement in the past 5 years, with exciting consequences for the diagnosis, treatment and follow‐up of surgical patients.

A series of enabling technologies and projects have expanded the knowledge of how basic molecular biology can assist in the management of surgical disease. The first, and most important, was the Human Genome Project, established in 1990 by the US National Institutes of Health and the UK Sanger Centre^1^. This established the reference human genome by carrying out sequencing of multiple fragments of a reference human genome using the dye‐terminator technique described by Sanger and colleagues^2^. A consequence of this technology is that the project took 10 years to produce a single genome and cost over US $3 billion to complete.

The development of microarray technology and next‐generation sequencing (NGS) within the past 5 years has led to a step‐change in the implementation of genomic technologies; before this, the bulk of genetic research was carried out on DNA microarrays.

### Genome‐wide association studies

**Figure 1:**
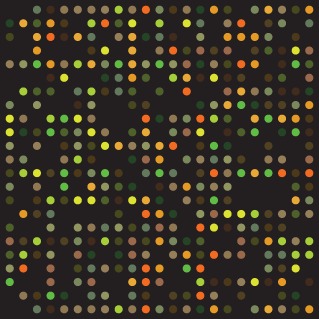
Image of scanned oligonucleotide array (from Wikimedia Commons)

**Figure 2:**
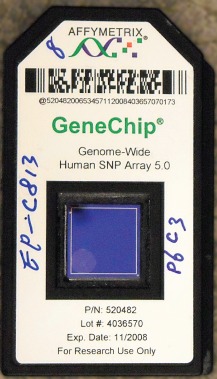
Affymetrix microarray chip from Wikimedia Commons

DNA microarrays are available from a variety of manufacturers (Illumina, Affymetrix and Agilent) and consist of silicon or glass slides with oligonucleotides complementary to the DNA sequence being studied, which are annealed to their surface. This allows cheap, mass production of microarrays that can be used for large population‐based studies. Typically these microarrays have between 500 000 and 1·5 million genomic markers, usually single‐nucleotide polymorphisms (SNPs). SNPs are single‐nucleotide changes within a gene that lead to protein change and subsequent change in the function of that gene. When scanned with a laser, each individual oligonucleotide fluoresces a specific colour, depending on the bound oligonucleotide fragment (*Figs* 1 and 2).

A variety of projects have been undertaken using DNA microarrays, typically taking the form of the genome‐wide association study (GWAS). These are usually case–control studies with cases enriched for the disease of interest. SNPs of interest are identified and taken forward to validation in larger cohorts, giving insights into the disease process being studied. Examples of GWASs include the COGENT (COlorectal cancer GENeTics) Consortium, and the Wellcome Trust Case Control Consortium 1/2 (WTCCC 1/2) examining colorectal cancer, Crohn's disease, diabetes, ischaemic heart disease, and several other common diseases and pathologies.

### Next‐generation sequencing

**Figure 3:**
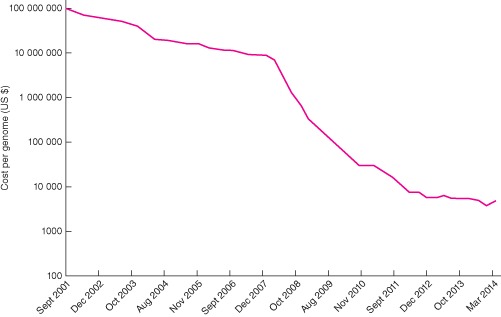
Cost in US dollars per genome sequenced in 2001–2014, at 5‐month intervals (data from http://www.genome.gov/sequencingcosts/)

The most widely used NGS technology, sequencing by synthesis (Illumina, San Diego, California, USA) allows entire human genomes to be sequenced within 24 h at low cost, with the sub $1000 genome barrier (X‐prize) being achieved earlier this year. Other innovative technologies include single molecular real‐time sequencing (PacBio® SMRT™; Pacific Biosciences, Menlo Park, California, USA), semiconductor sequencing (Ion Torrent™; Life Technologies, Paisley, UK) and nanopore sequencing (Oxford Nanopore, Oxford, UK). Until recently, NGS was limited to small studies on a few samples owing to cost constraints, but because of the rapid fall in price‐per‐sample (*Fig.* 3), large studies are now in progress. The Cancer Genome Atlas (TCGA) project is sequencing the cancer genome of 33 different cancer types in the US population; the 100 000 Genomes Project in the UK is undertaking to sequence 50 000 cancer genomes and 50 000 rare disease genomes over the next 5 years.

To carry out NGS, an inherently massively parallel technique, several steps are required. All technologies require capture of DNA or RNA into sequencing libraries. Illumina Solexa™ then uses this captured DNA/RNA to generate clusters, which are amplified. Cluster amplification is the process whereby target DNA is immobilized on to spatially separated template sites, allowing sequencing reactions to occur in parallel. Sequencing is then carried out on the clusters present on the glass slide. The slide is portioned into eight channels, enabling independent samples to be run simultaneously. Typically, reads of between 75 and 100 base pairs are possible, and the nucleotide incorporation cycle is repeated until sufficient depth is covered for all targeted regions. Data generated are then aligned with a reference sequence, and variants are called after comparing the sequencing data to a reference. This allows a tumour specimen to be compared with its paired normal tissue sample.

A critical difference in analysis of human cancers is the concept of germline and somatic mutations. Germline mutations are the constitutive DNA that the patient is born with, and variation within the germline confers an increased (or decreased) risk of cancer. Somatic mutations occur as a consequence of tumour development, although they may initiate tumour development by occurring spontaneously as a result of external factors such as ionizing radiation or carcinogens.

Microarray and sequencing technologies also allow analysis of other types of genetic information, such as DNA methylation (epigenetics), which acts as a switch in the regulation of gene expression. NGS also allows analysis of gene expression by NGS of mRNA (RNA‐seq) and other genetic modifications, such as sequencing of chromatin‐immunoprecipitated DNA (ChIP‐seq). Non‐coding small RNAs that may affect gene function, such as long non‐coding RNA (lncRNA), small interfering RNA (siRNA) and small nucleolar RNA (snoRNA), can also be analysed by NGS.

### Other 'omics technologies

Other technologies are emerging as potential methods for the downstream analysis and stratification of patient samples in surgical disease. Two examples of these technologies are metabolomics and proteomics. Metabolomics uses either nuclear magnetic resonance or mass spectrometry to ascertain the presence of metabolites in surgical specimens. The patterns and relative abundances of the metabolites observed can give clues as to the underlying biological processes at work in the tissues studied^3^.

Proteomics uses mass spectrometry to understand the structure of proteins. Several methods exist to allow proteins to be studied in the ionized form without fragmentation or damage including matrix‐assisted laser desorption ionization (MALDI)^4^ and electrospray ionization^5^. Because proteins are modified after they are produced by transcription (post‐translational modification), study of both the protein and RNA involved in tissues allows a fuller appreciation of the changes that may be occurring in a particular disease.

These technologies have allowed advances in understanding of the initiation and progression of multiple surgical diseases, and in adjuvant therapies for surgical disease such as chemotherapy and radiotherapy. They also allow the possibility of population screening of asymptomatic carriers.

## Lower gastrointestinal tract: colorectal cancer and inflammatory bowel disease

### Genetic predisposition

The predominant surgically relevant disease types studied in the lower gastrointestinal (GI) tract have been colorectal cancer, Crohn's disease and ulcerative colitis. The COGENT Consortium^6^ has undertaken multiple GWASs of patients with colorectal cancer associated with a strong family history or extreme phenotype (such as young age of onset), identifying ten SNPs associated with colorectal cancer at population‐wide significance. Although inheritance of one disease association SNP confers a population risk of odds ratio approximately 1·05 (*Table* 1, taken from Tenesa and Dunlop^7^), inheritance of multiple SNPs (more than 10) confers a cumulative threefold risk of cancer. Other successes from the GWAS/NGS approach have been identification of the *GREM*‐associated duplication in hereditary mixed polyposis syndrome^8^ and mutations in the DNA polymerase genes *POLE* and *POLD* as a cause of hereditary polyposis^9^. Identification of these mutations allows familial testing, enhanced surveillance and reduction in the risk of developing colorectal cancer.

**Table 1:** Frequency of identified single‐nucleotide polymorphisms in colorectal cancer, and their effect sizes (from Tenesa and Dunlop^7^)

Gene/locus	Chromosome	SNP	Effect size (odds ratio)	Allele frequency	Population attributable risk (%)
–	8q24	rs6983267	1·21 (1·15, 1·27)	0·51	9·7
*GREM1*	15q13	rs4779584	1·26 (1·19, 1·34)	0·18	4·5
*SMAD7*	18q21	rs4939827	1·18 (1·12, 1·23)	0·52	8·6
–	11q23	rs3802842	1·12 (1·07, 1·17)	0·29	3·4
*EIF3H*	8q23	rs16892766	1·25 (1·19, 1·32)	0·07	1·7
—	10p14	rs10795668	1·12 (1·10, 1·16)	0·67	7·4
*BMP4*	14q21	rs4444235	1·11 (1·08, 1·15)	0·46	4·8
*CDH1*	16q22	rs9929218	1·10 (1·06, 1·12)	0·71	6·6
*RHPN2*	19q13	rs10411210	1·15 (1·10, 1·20)	0·90	11·9
*BMP2*	20q12	rs961253	1·12 (1·08, 1·16)	0·35	4·0

Values in parentheses are 95 per cent c.i. SNP, single‐nucleotide polymorphism.

In Crohn's disease, multiple large‐population GWAS studies^10^ have been undertaken identifying multiple SNPs of predisposition, suggesting that Crohn's disease has a strong heritable component. In total, more than 73 SNPs have been identified, with the strongest association in the *NOD2* gene, which plays an important role in immunity. In total, these loci make up about 20 per cent of the observed inheritability of Crohn's disease.

Comparatively less research has been undertaken in germline susceptibility to ulcerative colitis; several large population GWAS studies^11^, ^12^, ^13^ have demonstrated over 30 associated SNPs. These SNPs are in a variety of genes, but are associated predominantly with immune system and immunity‐related genes. In addition, approximately 50 per cent of identified loci overlap with those of Crohn's disease.

### Genomic analysis of colorectal cancer

The colorectal cancer TCGA project^14^ has carried out exome sequencing (sequencing of the protein coding regions of the genome), RNA‐seq, genome‐wide methylation analysis and protein expression (via reverse‐phase protein arrays; RPPAs) of, at the time of writing, 461 colorectal tumours. This group has confirmed recurrent driver mutations in *APC*, *TP53*, *SMAD4*, *PIK3CA* and *KRAS*, but also found novel therapeutic targets in *ARID1A*, *SOX9* and *FAM123B*. Another study^15^ observed gene fusions (merging of two genes, which causes abnormal function) in R‐spondin. The mutations observed in *ARID1A* are particularly exciting as they present a potential therapeutic target^16^. These data sets provide a wealth of information about colorectal cancer, and linkage to a clinical data set provides opportunities for future biomarker studies.

Recent work has examined the role of integration of multiple 'omics data sets to produce classifiers of disease^17^, also known as endotypes. These are based on mutation, expression and immunological data sets. The Colorectal Cancer Subtyping Consortium found four distinct Colorectal cancer Molecular Subtypes (CMSs) (*Table* 2). The classifiers identified provide insight into the biology of the distinct types. CMS1 consisted of microsatellite‐unstable, immunologically active tumours occurring mainly on the right side in the elderly, whereas CMS2 (the most frequent endotype) consisted of chromosomally unstable, microsatellite‐stable tumours. Further study of these classifiers may permit finer stratification, allowing precisely targeted therapy.

**Table 2:** The Colorectal Cancer Subtyping Consortium classification of colorectal cancer

Classifier	Frequency (%)	Characteristics
CMS1	14	MSI, immune pathway activation/expression, right‐side tumours, older age at diagnosis, females, hypermutation, *BRAF* mutation, intermediate survival
CMS2	41	High CIN, MSS, strong Wnt/Myc pathway activation, left‐side tumours, *TP53* mutation, *EGFR* amplification/overexpression, better survival
CMS3	8	Low CIN, moderate Wnt/Myc pathway activation, *KRAS* mutation, *PIK3CA* mutation, *IGFBP2* overexpression, intermediate survival
CMS4	20	CIN/MSI heterogeneous, mesenchymal/TGF‐β activation, younger age at diagnosis, *NOTCH3*/*VEGFR2* overexpression, worse survival

CMS, Colorectal cancer Molecular Subtype; MSI, microsatellite instability; CIN, chromosomal instability; MSS, microsatellite stable; TGF, transforming growth factor.

### Screening biomarkers

A wide variety of biomarkers have been examined^18^ in colorectal cancer, as both markers of screening and of prognosis. The ideal biomarker would be easily detectable in either stool or blood, cheap and highly accurate. Unfortunately, no current biomarker fits these criteria precisely owing to the molecular heterogeneity associated with colorectal cancer. The most promising markers seem to be associated with abnormal DNA methylation. For example, in colorectal cancer, differential methylation of the septin 9 gene has been shown to have 72 per cent sensitivity and 90 per cent specificity for the detection of malignancy. However, many biomarker studies across all cancer types are plagued by poor study design, insufficient power and non‐hypothesis‐driven marker selection. Currently, a well designed, UK‐based trial of methylated biomarkers, the ENDCaP‐C study (Enhanced Neoplasia Detection and Cancer Prevention in Chronic Colitis) is under way^19^, examining their value in the detection of dysplasia in a screened population of patients with ulcerative colitis.

Another rich field of developing interest in colorectal cancer is sequencing of the microbial genomes that exist within the colon. Experimental murine models seem to indicate that the microbiome within the colon alters the risk of colorectal cancer by modulating inflammation^20^. This has also been demonstrated to be the case in patients with inflammatory bowel disease, for both ulcerative colitis and Crohn's disease^21^.

Metabolomic techniques also show promise in acting as screening biomarkers. Mirnezami and co‐workers^22^ undertook high‐resolution magic‐angle spinning nuclear magnetic resonance spectroscopy in 44 tumour–normal pairs, finding cancer‐specific metabolite patterns allowing differentiation of cancer from normal tissues, in addition to finding changes in the metabolome as the tumour progressed. A number of proteomic studies have also been carried out in colorectal cancer^23^, although these all suffer from lack of validation, and the variety of different markers identified undoubtedly reflects the varying populations from which they were sampled.

### Targeted therapies, prognostic and predictive biomarkers

The discovery that the epidermal growth factor (EGF) pathway in colorectal cancer was sensitive to inhibition by anti‐EGF receptor (EGFR) monoclonal antibodies (mAbs) led to the rapid development of panitumumab and cetuximab. However, it was found in initial trials that the antibodies seemed to have no clinical effect against colorectal cancer; although a proportion of patients seemed to benefit from therapy, the majority did not^24^. It was found subsequently that mutations in the EGFR pathway genes (*KRAS*, *BRAF*, *NRAS* and *PIK3CA*) conferred resistance to anti‐EGFR mAbs due to hyperactivation of the pathway independent of the EGFR^25^. The CRYSTAL trial^26^ compared patients with a *KRAS* mutation and those without, finding a clear response and survival benefit for anti‐EGFR mAbs in patients without mutation. A number of other pathway‐specific inhibitors exist for colorectal cancer, including the antivascular endothelial growth factor (VEGF) mAb bevacizumab, MEK inhibitors that target EGF pathway mutated cancers and cancer vaccines. The FOCUS4 trial^27^ is currently recruiting patients for a molecularly stratified trial of metastatic colorectal cancer therapy: patients are selected for a specific therapy when they possess a mutation specific to that cancer. This raises the intriguing possibility of molecular‐targeted therapy for primary, non‐metastatic tumours as neoadjuvant therapy before surgery.

A commercially available test exists for prediction of recurrence and benefit for 5‐fluorouracil chemotherapy (Oncotype DX®; Genomic Health, Redwood City, California, USA), based on a multigene panel of RNA expression from formalin‐fixed paraffin‐embedded tissues^28^. This was developed by screening 761 candidate genes against a large cohort of 1851 patients undergoing surgery for colorectal cancer, with or without adjuvant 5‐fluorouracil therapy. Validation was carried out on the QUASAR study^29^, which could stratify prognosis, but did not correlate with benefit from chemotherapy.

Another intriguing possibility is the use of immune‐based stratification to estimate colorectal cancer prognosis. Lal *et al*.^30^ used the TCGA expression data set to identify four different immune classifiers based on the expression of immune system‐related genes. Immunogenicity is thought to be related to survival, as tumours that are more visible to the immune system are more likely to undergo destruction by the immune system. One of the mechanisms that may occur in highly mutated tumours, such as tumours with a *POLE* mutation or those with microsatellite instability, is where the large number of mutations causes a variety of frameshift mutations. These frameshift mutations drive the production of neoantigens^31^ caused by the alternative splicing of multiple genes, which increases the visibility of the tumour to the immune system.

## Upper gastrointestinal tract: oesophagogastric cancer

### Genetic predisposition

Research into predisposition to gastro‐oesophageal cancer is complicated by the fact that it can arise in two histologically different epithelial types: squamous cell carcinoma (SCC) and adenocarcinoma. A significant proportion of the risk is likely to comprise lifestyle factors such as smoking, gastro‐oesophageal reflux and diet.

In adenocarcinoma, a GWAS of the premalignant stage of oesophageal cancer^32^, Barrett's oesophagus, demonstrated associations between the major histocompatibility locus and a gene associated with oesophageal development (*FOXF1*). It was also found that the predisposition to Barrett's oesophagus was made up of multiple common variants of small effect, rather than a single genetic driver. A further GWAS of oesophageal adenocarcinoma^32^ demonstrated associations with transcription factors (*CRTC1*, *FOXP1*, *BARX1*). In light of these results it is difficult to highlight SNPs that may act as markers for increased disease risk or act as molecular targets for therapy.

Several GWAS studies have been undertaken, predominantly in Chinese populations at high risk of oesophageal SCC^33^, ^34^. They highlighted SNPs in the riboflavin transporter *C20orf54*, and a cell growth and differentiation gene, *PLCE1*. Riboflavin deficiency was identified before this study as a risk factor^35^ for oesophageal SCC. The *PLCE1* variant was further found to interact specifically with tobacco smoke exposure^36^.

### Genomic analysis of oesophagogastric cancer

Both the Broad Institute (Cambridge, Massachusetts, USA) and the OCCAMS (Oesophageal Cancer Clinical and Molecular Stratification) Consortium have studied oesophageal adenocarcinoma. The Broad Institute project^37^ undertook whole‐exome and whole‐genome sequencing in 149 tumour–normal pairs, verifying previously identified mutations in *TP53*, *CDKN2A*, *SMAD4*, *ARID1A* and *PIK3CA*. Previously unidentified mutations in *SPG20*, *TLR4*, *ELMO1* and *DOCK2* were also found, and a possible role for the RAC1 pathway (a modulator of epithelial–mesenchymal transition) was identified.

The OCCAMS Consortium^38^ examined whole‐genome sequencing of oesophageal adenocarcinoma, as well as targeted sequencing of never‐dysplastic Barrett's oesophagus (which did not progress to malignancy) and high‐grade dysplasia. They found that *TP53* was the dominant mutation seen in adenocarcinoma, in over 80 per cent of samples, but that these mutations were also present in biopsies from never‐dysplastic patients, contrary to what was expected based on the known oncological progression of these lesions. The only stage‐specific mutations seen in high‐grade dysplasia and adenocarcinoma were in *TP53* and *SMAD4*.

Whole‐genome and whole‐exome sequencing of oesophageal SCC has also been performed^39^. Whole‐genome sequencing of 17 tumour–normal pairs and whole‐exome sequencing in a further 71 tumour–normal pairs identified recurrent mutations in *TP53*, *RB1*, *CDKN2A*, *PIK3CA*, *NOTCH1* and *NFE2L2*, as well as *ADAM29* and *FAM135B*. The genomic landscape of oesophageal SCC was significantly different from that of oesophageal adenocarcinoma, highlighting the different therapeutic strategies that are needed in this disease.

### Biomarkers of predisposition/sensitivity and screening

Given the above OCCAMS findings, it is difficult to use *TP53* mutation as a biomarker of adenocarcinoma, as it has also been identified in biopsies from never‐dysplastic patients, who should not progress to adenocarcinoma. As a precursor to the OCCAMS study^38^, the same group undertook combined‐array CGH (comparative genomic hybridization, a type of microarray analysis looking at chromosomal abnormalities) in tumour samples and gene expression via microarray^40^. They found a pattern of copy number alterations and associated expression change that could identify poor‐prognosis oesophageal adenocarcinoma. The OCCAMS group also examined gene expression changes in adenocarcinoma using RNA microarrays^41^, finding a four‐gene expression panel of *DCK*, *PAPSS2*, *SIRT2* and *TRIM44* that were independently predictive of survival.

There have been a number of attempts at developing methylated biomarkers in oesophageal adenocarcinoma, both as screening biomarkers and to identify high‐risk Barrett's oesophagus. The genes studied include *CDKN2A*, vimentin, *P14ARF*, *CDX2*, *SOCS1/3*, *SFRP1/2/4/5* and *WIF1* 42, 43, 44. Unfortunately no consistent marker can be identified that successfully differentiates adenocarcinoma from normal oesophagus and high‐grade Barrett's dysplasia. These types of focused biomarker will become increasingly important to stratify therapy.

Attempts have been made using proteomics^45^ to distinguish oesophagogastric cancer from benign disease, to allow screening. MALDI mass spectrometry was used to compare the differences between oesophageal cancer and normal mucosa, and gastric cancer and normal mucosa. It was found that, although there were clear differences between cancer and normal tissue, there was a wide variety of changes that varied between different tumours, making determination of a specific biomarker difficult.

### Targeted therapies

Despite the recent advances in discovery science in gastro‐oesophageal cancer, few therapeutic targets currently exist. A small proportion of cancers overexpress the human EGFR 2 (HER2) protein^46^, and clinical trials of trastuzumab, a mAb against the HER–receptor complex are ongoing. The ToGA (Trastuzumab for Gastric Cancer) study^47^ investigated the addition of trastuzumab to standard chemotherapy, and demonstrated a small survival benefit in HER2‐positive cancer. Targeting by anti‐VEGF therapy is also undergoing clinical trials^48^; however, results have been mixed with no improvement in overall survival, but improvements in response rates and progression‐free survival.

## Breast cancer

### Genetic predisposition

The role of inherited variability in breast cancer has been investigated extensively. A very strong signal for variants in the fibroblast growth factor receptor 2 gene (*FGFR2*) have been found in GWAS studies across multiple populations^49^, ^50^, ^51^. Carriers of the two low‐risk alleles at *FGFR2* (frequency 38 per cent of the population) have a relative risk of breast cancer of 0·83 compared with the general population^52^; carriers of one high‐risk and one low‐risk allele (47 per cent) have a relative risk of 1·05; and carriers of two high‐risk alleles (14 per cent) have a relative risk of 1·26^53^. *FGFR2* mutations are of particular interest as they may represent a therapeutic target in breast cancer^54^.

A recent GWAS^55^ in oestrogen receptor (ER)‐negative breast cancer demonstrated four variants that reached genome‐wide significance in *MDM4*, *LGR6*, *FTO* and a SNP within the 2p24·1 region. These SNPs were present only in ER‐negative breast cancers, in contrast to the findings in a combined ER‐positive/ER‐negative GWAS^56^. This found variants located with *PTHLH*, known to have a role in breast development, and *NRIP1*, a co‐factor of the ER.

### Next‐generation sequencing

A number of NGS projects have examined the mutation spectrum in breast cancer. The most comprehensive of these is from the TCGA project^57^, which carried out exome sequencing, RNA‐seq, methylation array analysis and RPPA of 463 patients. Recurrent mutations were found in *TP53*, *PIK3CA* and *GATA3* at frequencies of over 10 per cent, reinforcing their role as driver mutations in breast cancer, as well as mutations in several dozen genes previously identified in breast cancer. Study of the role of expression subtypes in breast cancer demonstrated four separate subtypes (luminal A/B, basal and HER2E), with the mutational burden and spectrum varying in each. The HER2E subtype demonstrated a relatively low mutational frequency, whereas the luminal A subtype demonstrated large numbers of significantly mutated genes, the most frequent mutation being in *PIK3CA*.

In common with colorectal cancer, methylation array analysis of breast cancer revealed a hypermethylator phenotype, associated with the luminal B expression subtype, and a hypomethylated phenotype associated with a basal expression subtype and comparatively higher frequency of *TP53* mutation. Copy number analysis was also performed, with the previously identified amplifications in *HER2* and *EGFR* being identified, as well as novel amplifications in *PIK3CA* and *FOXA1* and deletions in *RB1* and *PTEN*. A striking finding throughout the study was the detection of genetic heterogeneity both within tumours and between samples, highlighting the diverse nature of this disease.

Shah and colleagues^58^ examined the mutational spectrum in triple‐negative breast cancer (ER/progesterone receptor/HER‐negative), again finding that *TP53* and *PIK3CA* mutations were clonally dominant, but also finding a wide variety of mutation spectra, including tumours with few driving mutations and tumours with extremely complex mutational spectra (the hypermutated phenotype). A separate study^59^ identified recurrent mutations in the transcription factor genes *CBFB* and *RUNX1*, as well as a gene fusion seen only in triple‐negative breast cancers, the *MAGI3–AKT3* fusion transcript. An exome sequencing study^60^ of 100 breast cancers at the Wellcome Trust Sanger Institute (Cambridge, UK) identified more than 40 driver mutations in a breast cancer cohort, including genes now known to be important therapeutic targets, such as *AKT1*/*2*, *ARID1B*, *CASP8* and *MAP3K1*.

NGS also has applications in the monitoring of metastatic disease or in recurrence. Dawson *et al*.^61^ used a combination of targeted NGS, digital PCR and whole‐genome sequencing to examine DNA circulating in the bloodstream that is shed from metastatic tumours. They found that increasing amounts of circulating DNA correlated with poorer overall survival, but also that, by comparing mutations in the primary tumour with the cell‐free DNA obtained, recurrence of the primary tumour could be detected by the presence of the same somatic mutations in the circulating DNA. This technology has applications for the detection of recurrence in multiple tumour types across the disease spectrum.

### Biomarkers of predisposition/sensitivity and screening

One‐step nucleic acid amplification (OSNA) is an example of the use of genetic technologies to stratify patients. It works by the detection of raised copy number of the cytokeratin 19 gene (*CK19*) as a surrogate marker of the presence of breast cancer within sentinel lymph nodes^62^, and is used as a proxy marker for axillary lymph node positivity in breast cancer. OSNA has been approved as a technological solution to determine sentinel lymph node positivity by the UK National Institute for Health and Care Excellence (NICE), and has been shown to be equivalent to both radioisotope‐ and dye‐based technologies for mapping lymph nodes^63^.

Another recent development is the DNA damage response assay for the prediction of response to anthracycline/cyclophosphamide‐based chemotherapy in breast cancer^64^. The study examined RNA expression in patients with a DNA damage response deficiency and developed a 44‐gene RNA expression panel that could predict response to chemotherapy in sporadic breast cancer.

### Targeted therapies

A rich variety of targeted therapies exist for breast cancer, based on the underlying biology of the tumour, gained by the extensive molecular research carried out on breast cancer. Aromatase inhibitors (such as anastrazole) and targeted ER modulators (such as tamoxifen) have been used extensively for many years, based on the observation that a proportion of breast cancers are ER‐positive. Identification of the overexpression of HER2 in breast cancer has allowed targeting with both mAbs (trastuzumab) and small‐molecule tyrosine kinase inhibitors such as lapatanib, with appreciable survival benefits.

More recently, several novel pathways have been identified as being dysregulated in breast cancer: the insulin‐like growth factor (IGF) 1 pathway; the *BRCA*‐associated double‐strand break repair protein, poly(ADP‐ribose) polymerase (PARP) 1; and the phosphatidylinositol‐3‐kinase (PIK3)–Akt–mammalian target of rapamycin (mTOR) pathway. Immunohistochemistry of IGF‐1 has revealed that it is overexpressed in more than 50 per cent of breast cancers, and a mAb (cixutumumab) that targets these pathways is currently in phase 1 studies^65^.

PARP inhibitors were identified from work targeting *BRCA* mutant breast cancer^66^ as a therapeutic strategy to treat patients with *BRCA* mutations. A small molecular PARP inhibitor, olaparib, has been used to treat germline *BRCA* mutant breast cancer^67^, with a clinical trial (the OlympiAD trial) currently under way. PARP inhibitors also show promise in triple‐negative breast cancer, as studies have shown that a proportion of these women possess *BRCA* mutations^68^, ^69^ and may respond to PARP inhibitors. mTOR pathway inhibitors, such as everolimus, should theoretically be of benefit in breast cancer as a result of the dysregulation of this pathway demonstrated by molecular studies, although initial results have been disappointing^70^.

## The future

The union of basic biological research with surgery has allowed the field of translational surgical biology to develop, utilizing modern molecular technologies to stratify surgical disease based on therapeutic and outcome response.

A number of possibilities exist for future research. First, whole‐genome studies using NGS technologies of prospectively collected samples will allow identification of biomarkers for response (such as radiotherapy in rectal cancer) and treatment (for instance, molecularly targeted therapies). An example of current clinical issues that could be answered using this technology is the response of rectal cancer to radiotherapy. Around 5–10 per cent of patients with advanced rectal cancer given preoperative radiotherapy have a complete response^71^. Research is currently under way to understand what makes these tumours particularly sensitive (the so‐called extreme responders) to radiation, and it is likely that a highly focused approach using NGS will identify responsible pathways.

Stratification of premalignant lesions is also an important focus of research. In colorectal, upper GI and breast cancer, few biomarkers exist to predict which premalignant lesions will progress to invasive cancer. Typically less than 1 per cent of patients with Barrett's oesophagus will progress to invasive oesophageal cancer, but no markers reliably predict this. The use of retrospective cohorts of patients with progressive Barrett's oesophagus and NGS analysis might identify genetic markers.

The concept of disease classifiers will play an increasingly important role and, as more NGS data sets become available, the granularity of classifiers will improve to the extent whereby a more precise understanding of the biology underlying a tumour will be available. This will be enhanced by the integration of multiplatform (NGS, metabolomics, proteomics) data into these classifiers, and will allow tailoring of therapy to the underlying disease.

The availability of NGS data sets for the surgical population will be enhanced by the commissioning of the UK 100 000 Genomes Project^72^. This exciting project will carry out whole‐genome sequencing of 50 000 tumour–normal pairs from patients with a range of cancers, but concentrating primarily on colorectal, breast, lung and prostate cancer. The data made available by this project will allow a highly detailed examination of the drivers in these cancer types; this is the largest project of its type in the world.

Although the technological advances are numerous, they are not without their own challenges. The recent identification of intratumoral heterogeneity, long hypothesized but only recently identified definitively in renal cell cancer^73^ and in preneoplastic lesions^74^, provides unique challenges for stratification. It is likely that multiple subclones of tumour exist within a primary tumour, each with differing characteristics. These will dictate prognosis and response to therapy, and further investigation is needed to understand the extent and consequences of this phenomenon.

Technological leaps in molecular biology will enable selection of the right therapy for the right patient at the right time, and further build on the surgical and anaesthetic improvements achieved in the past century to provide maximal benefit for the patient.
